# In-Hospital Mortality and Glycemic Control in Patients with Hospital Hyperglycemia

**DOI:** 10.1900/RDS.2021.17.50

**Published:** 2021-10-31

**Authors:** María Paula Russo, Santiago Nicolas Marquez Fosser, Cristina María Elizondo, Diego Hernán Giunta, Nora Angélica Fuentes, María Florencia Grande-Ratti

**Affiliations:** 1 Internal Medicine Research Unit, Hospital Italiano de Buenos Aires, Buenos Aires, Argentina,; 2 Clinical and Health Informatics Research Group, McGill University, Montréal, Québec, Canada,; 3 Department of Health Informatics, Hospital Italiano de Buenos Aires, Ciudad de Buenos Aires, Argentina,; 4 Centro de Investigaciones Clínicas, Mar del Plata, Argentina.

**Keywords:** hospital hyperglycemia, hospital mortality, stress hyperglycemia, hospital hyperglycemia

## Abstract

**BACKGROUND:**

Stress-induced hyperglycemia is a phenomenon that occurs typically in patients hospitalized for acute disease and resolves spontaneously after regression of the acute illness. However, it can also occur in diabetes patients, a fact that is sometimes overlooked. It is thus important to make a proper diabetes diagnosis if hospitalized patients with episodes of hyperglycemia with and without diabetes are studied.

**AIMS:**

To estimate the extent of the association between stress-induced hyperglycemia and in-hospital mortality in patients with hospital hyperglycemia (HH), and to explore potential differences between patients diagnosed with diabetes (HH-DBT) and those with stress-induced hyperglycemia (SH), but not diagnosed with diabetes.

**METHODS:**

A cohort of adults with hospital hyperglycemia admitted to a tertiary, university hospital in Buenos Aires, Argentina, was analyzed retrospectively.

**RESULTS:**

In the study, 2,955 patients were included and classified for analysis as 1,579 SH and 1,376 HH-DBT. Significant differences were observed in glycemic goal (35.53% SH versus 25.80% HH-DBT, p < 0.01), insulin use rate (26.66% SH versus 46.58% HH-DBT, p < 0.01), and severe hypoglycemia rate (1.32% SH versus 1.74% HH-DBT, p < 0.01). There were no differences in hypoglycemia rate (8.23% SH versus 10.53% HH-DBT) and hospital mortality. There was no increase in risk of mortality in the SH group adjusted for age, non-scheduled hospitalization, major surgical intervention, critical care, hypoglycemia, oncological disease, cardiovascular comorbidity, and prolonged hospitalization.

**CONCLUSIONS:**

In this study, we observed better glycemic control in patients with SH than in those with HH-DBT, and there was no difference in hospital mortality.

## 1 Introduction

Among hospitalized patients, both hyperglycemia and hypoglycemia are associated with adverse outcomes, including death [1, 2]. Hospital hyperglycemia (HH), defined as blood glucose values higher than 140 mg/dl (7.8 mmol/l) according to current guidelines, is an important in-hospital problem [3]. Blood glucose levels persistently above this level should prompt conservative interventions, such as alterations in diet or changes in the medication that causes hyperglycemia. The prevalence of HH is variable according to published articles, but is approximately 40% [4-6]. Patients with HH may have a diagnosis of diabetes or their HH could be an isolated case of stress-induced hyperglycemia (SH) [7].

HH is not usually the main problem during hospitalization. Therefore, it is common for glycemic control in patients diagnosed with diabetes or SH to be lost within the problem list [8]. Individualized glycemic control goals in hospitalized patients with HH are based on studies conducted in critical care areas, and do not take into account previous diagnosis of diabetes. There is no adequate evidence on how to follow up patients with HH in non-critical care areas [9-11].

Several articles have concluded that SH is a predictor of increased morbidity and mortality during hospitalization [12, 13]. In this regard, other articles propose that a diabetes diagnosis is also associated with morbidity and mortality during hospital stay [14-16].

The aim of this article is to describe the association between SH and hospital mortality in patients with HH. Also, it aims to describe potential differences in glycemic control between patients with SH and those with HH who have been previously diagnosed with diabetes (HH-DBT).

## 2 Methods

### 2.1 Study design

A retrospective cohort study of hospitalized patients with hospital hyperglycemia was conducted in 2014-2015 in a highly complex university hospital, in Buenos Aires, Argentina. Hospital Italiano de Buenos Aires deals with 2,900,000 outpatient visits every year, 46,000 inpatients, and 43,500 surgical procedures in its 41 operating rooms. Its hospital capacity reaches 750 beds, with 200 beds in critical care units.

All adult patients referred to the hospital between the period 06/01/2014 and 05/31/2015 for any clinical or surgical cause, who presented hospital hyperglycemia (HH), defined as at least one value over 140 mg/dl, were included in this study. Pregnant women were excluded as they have different blood glucose values for the current definition of glucose disorders. Likewise, patients classified as undetermined hyperglycemia (without a previous diagnosis of diabetes or in-hospital HbA1c values) were excluded.

If patients had more than one hospitalization during the study period, then only the first one was included. For unscheduled hospitalizations, the hospitalization start date was defined as the time of admission to the emergency department; otherwise, it was the date of admission for scheduled hospitalizations. All patients were followed until discharge or in-hospital death.

All patient healthcare information at the hospital is stored in a clinical data repository (CDR), which has been operating for more than 10 years. The CDR has mirrored databases with non-identifying information to ensure privacy and confidentiality. To enable secondary analysis, the CDR was used as an information source where are all clinical documents are deposited from different sources such as test results, images, clinical notes, drug prescriptions, pharmacy dispensations, outpatient visits, emergency department visits, inhospital care, and others. Therefore, the information in the patient cohort for the present study was collected from one of these high-quality secondary healthcare databases in the hospital network and integrated into the electronic health records (EHR) using a relational database model, in which data are stored in tables that allow one-to-one, one-to-many, and many-to-many relationships. Data from the secondary databases were obtained from the EHR; all glycemic and HbA1c values during hospitalization and from the 3 months before and/or after hospital discharge were retrieved.

### 2.2 Data processing

The data was stored in an encrypted Access database, exclusively available to researchers involved in the study. The data were processed using Microsoft Access 2007.

### 2.3 Definitions and subgroup classification

Hospital hyperglycemia (HH) was defined as at least one blood glucose value >140 mg/dl during hospitalization. Altered HbA1c was defined as at least one value ≥7% or two values ≥6.5% prior to admission. Altered hospital HbA1c was defined as at least one value ≥6.5% recorded in the EHR during hospitalization or up to 3 months after discharge.

These definitions were used to classify patients into 2 subgroups of hyperglycemia:

1. Diabetic patient (HH-DBT): when a diabetes-related problem was registered upon admission to the hospital or when the patient had HH determined by high previous, in-hospital, or post-discharge HbA1c values.
2. Patients with stress hyperglycemia (SH), but without diabetes: HH without a diabetes diagnosis and normal HbA1c values (pre-, post-, or in-hospital).

A patient was considered as a critical care patient if during hospitalization a stay in an intensive care unit (ICU) or coronary care unit (CCU) was required. A patient’s hospitalization was defined as a surgical intervention if a major surgery was required. It was classified as an unscheduled hospitalization when the patient entered through the emergency department. The main hospital service used for the patient was defined based on which specialist provided the main attention required. This was classified into four groups:

1. Cardiology: cardiology and cardiovascular surgery
2. Internal medicine: internal medicine, hepatology, neurology, psychiatry, oncology, hematology, and dermatology
3. Critical care: ICU and CCU
4. Surgery: general surgery, urology, traumatology, otorhinolaryngology, and ophthalmology

Cardiovascular comorbidities were defined as a background of acute myocardial infarction, stroke, transient ischemic attack, heart failure, peripheral vascular disease, or chronic renal failure. Oncological diseases were defined as a background of solid malignant tumor, leukemia, or lymphoma.

As per glycemic records, we analyzed electronic glycemic assessments made during the in-hospital period, i.e. venous glycemic analysis and capillary monitoring. The following were defined:

- Glycemic goal (GG): median glucose value ≤180 mg/dl (venous and capillary monitoring).
- Hypoglycemia: at least one glycemic value <70 mg/dl.
- Severe hypoglycemia: at least one glycemic value <40 mg/dl.

Regarding insulin use, patients were divided into three different groups based on the type of insulins used:

1. Short-acting insulin: aspart, lispro, glulisine or current.
2. Long-acting insulin: NPH insulin, detemir or glargine.
3. Long-/short-acting insulin: if both types of insulins were used.

Glycemic variability is emerging as a quality and safety indicator of glycemic control in the literature [17, 18]. We assessed glycemic variability in those patients with at least two in-hospital days and two glycemic values.

The following numerical variables were defined to assess glycemic variability:

- Interquartile range (subtraction between first and third percentile) [1,9]
- Delta blood glucose (the difference between the minimum and maximum value) [20]
- Average glucose value [2,1] - Standard deviation [22-26]
- Coefficient of variability (standard deviation / mean glycemic value) (CV) [27]

The coefficient of variation (CoV) was classified as excellent (<33.5%), good (33.5-36.8%), regular (36.8-40.6%), and bad (>40.6%) [27]. When the CoV was excellent, patients were considered as not presenting glycemic variability. All other values (good, regular, and bad) were considered as having glycemic variability.

### 2.4 Statistical analysis

Quantitative variables are presented as mean and standard deviation (SD) or median and interquartile range (IQR) according to the distribution found. The normality of data was verified through the Shapiro-Wilk or Kolmogorov-Smirnov test, as appropriate. Categorical variables are presented as absolute and relative percentage frequencies. Both groups (SH and HH-DBT) were compared to assess in-hospital mortality and glycemic goals using a chi-square test.

The raw and adjusted odds ratios (OR) were estimated using a logistic regression model. Confounders regarding clinical relevance and those that showed significant results in the raw analysis (p < 0.05) were included in the multivariate model. The ORs are reported with their confidence intervals. A statistical difference is considered significant if p < 0.05. The data were analyzed using the STATA program, version 13 (StataCorp, College Station, Texas-USA).

### 2.5 Ethical considerations

This article was approved by the hospital research ethics review committee. The national and international ethic regulatory standards, the World Medical Association, and the Helsinki declaration were respected. All data were treated in confidentiality and restricted access was granted only to researchers given the right to access personal data.

## 3 Results

**Figure 1.**
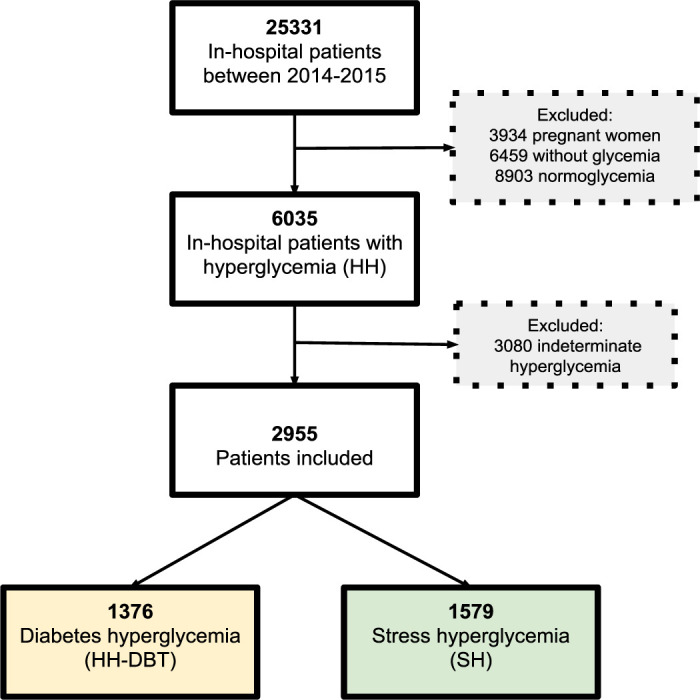
Flowchart of the patient selection process.

During the study period, 25,331 adult patients were hospitalized. Of those, 3,934 pregnant women, 6,459 patients without a glucose analysis, and 8,903 patients with normoglycemia during hospitalization were excluded. The number of patients with hospital hyperglycemia was 6,035. Of these, 3,080 patients classified as undetermined hyperglycemia were excluded due to the lack of hospital HbA1 records, leaving 2,955 patients that were included in the study (**Figure 1**).

Among the total of 2,955 patients with hospital hyperglycemia, the average age was 69.9 years (SD 15.10); 46.80% (1,383) were female, 56.82% (1,679) were admitted on an unscheduled basis, 47.21% (1,395) needed a critical care unit at some point during hospitalization, and 50.36% (1,488) required surgery. The median hospital stay was 5.9 days (IQR 7.9), and in-hospital mortality was 7.61% (225). Regarding the primary hospital service required, 37.53% (1,109) were assigned to internal medicine, 34.21% (1,011) to surgery (excluding non-cardiac surgery), 23.86% (705) to cardiology, and 4.40% (130) to critical care.

Of the 2,955 patients with HH, 46.56% (1,376) were diagnosed with diabetes and 53.44% (1,579) had stress-induced hyperglycemia without diabetes diagnosis. The demographic baseline and hospital characteristics of both groups are shown in **Table 1**.

**Table 1.** Patient characteristics

Characteristic	SH (n = 1,579)	HH-DBT (n = 1,376)	p-value
Age (yr.)^*^	72 (IQR 22)	71 (IQR 19)	0.236
Female	50.28% (794)	42.81% (589)	0.001
Critical care	52.18% (824)	41.50% (571)	0.001
Surgical intervention	54.84% (866)	45.20% (622)	0.001
Unscheduled hospitalization	54.91% (867)	59.01% (812)	0.025
Hospital service		
Cardiology	26.22% (414)	21.15% (291)
Internal medicine	35.66% (563)	39.68% (546)	0.009
Critical care unit	4.50% (71)	4.29% (59)
Surgery (non-cardiological)	33.63% (531)	34.88% (480)
Oncological disease	15.83% (250)	20.57% (283)	0.001
Cardiovascular comorbidities	36.61% (578)	47.17% (649)	0.001
Hospital stay (days)^*^	6.7 (IQR 8.9)	5.1 (IQR 6.94)	0.001

Legend:^*^ median and interquartile range. All other variables are absolute or percentage values.

Glycemic values in both groups are described in **Table 2**. Patients with SH had more glycemic value entries. Both groups reached the overall glycemic goal in a similar way, with 35.53% in SH (95% CI: 33.16-37.94%) and 25.80% in HH-DBT (95% CI: 23.50-28.19%), both p < 0.001.

**Table 2.** Glycemic values and behavior in both patient groups

Glycemic variable	SH (n = 1,579)	HH-DBT (n = 1,376)	p-value
Number of glycemic values^*^	8 (14)	6 (10)	0.001
Median glycemic values (mg/dl)^#^	124 (32)	155 (55)	0.001
Global glycemic goal 95% CI	35.53% (561/1579) 33.16-37.94	25.80% (355/1376) 23.50-28.19	0.001
Hypoglycemia	8.23% (130)	10.53% (145)	0.031
Severe hypoglycemia	1.32% (21)	1.74% (24)	0.359

**Legend:** All glycemic values are venous and capillary monitoring reports.

* mean (SD),

# median (interquartile range, IQR).

Patients with SH had a lower incidence of hypoglycemia, independent of the three insulin schemes used (basal, bolus, or basal-bolus). Severe hypoglycemia was a rare event, and there was no significant difference between both groups in this regard. When hypoglycemic values reported from venous samples only (i.e. non-capillarity measures) were considered, hypoglycemia in the HH-DBT group was more severe. **Table 3** describes the insulin treatment during hospitalization.

**Table 3.** In-hospital insulin treatment in both patient groups

Insulin treatment	SH (n = 1,579)	HH-DBT (n = 1,376)	p-value
Insulin use (total)	26.66% (421)	46.58% (641)	0.001
Short-acting insulin	83.37% (351)	62.71% (402)	0.001
Long-acting insulin	2.85% (12)	9.36% (60)	0.001
Short and long-acting insulin	13.78% (58)	27.92% (179)	0.001

An analysis of glycemic variability was carried out in 2,108 patients. There were differences in several glycemic variability parameters between the SH group (1,136) and the HH-DBT group (972). However, no difference was found regarding the glycemic variability coefficient, for which both groups show a low value. **Table 4** shows variables related to glycemic variability.

**Table 4.** Glycemic variability in both patient groups

Glycemic parameter	EH (n = 1,136)	HH-DBT (n = 972)	p-value
Maximum glycemic value	178 (IQR 67.5)	212 (IQR 106.5)	0.001
Minimum glycemic value	92 (IQR 22)	108 (IQR 42)	0.001
Delta blood glucose	85 (IQR 67)	98 (IQR 99.5)	0.001
Glycemic interquartile range	40 (IQR 33)	50 (IQR 50)	0.001
Glycemic SD	31.92 (IQR 19.87)	37.51 (IQR 33.01)	0.001
Coefficient of variability (SD/mean)	0.27 (SD 0.13)	0.27 (SD 0.14)	0.920

There was no significant difference in mortality between both groups, with 7.73% (95 % CI: 6.45-9.15) for SH and 7.49% (95% CI: 6.15-0.90) for HHDBT. Unadjusted OR mortality for SH was 1.03 (95% CI: 0.787-1.359, p = 0.805). Univariate analysis showed that the following variables were associated with a higher in-hospital mortality from SH: age, urgent hospitalization, critical care requirement during hospitalization, hypoglycemia, cardiovascular comorbidities, and long hospital stay >7 days (**Table 5**).

**Table 5.** Univariate analysis of in-hospital mortality

Glycemic variable	SH (n = 1,579)	HH-DBT (n = 1,376)	p-value
Number of glycemic values^*^	8 (14)	6 (10)	0.001
Median glycemic values (mg/dl)^#^	124 (32)	155 (55)	0.001
Global glycemic goal 95% CI	35.53% (561/1579) 33.16-37.94	25.80% (355/1376) 23.50-28.19	0.001
Hypoglycemia	8.23% (130)	10.53% (145)	0.031
Severe hypoglycemia	1.32% (21)	1.74% (24)	0.359

Legend:* In patients eligible for glycemic variability.

Regarding multivariate analysis, the mortality OR for the SH group adjusted by age, unscheduled hospitalization, major surgical intervention, critical care, hypoglycemia, cancer disease, cardiovascular comorbidities, and hospital stay >7 days was 1.11 (95% CI: 0.83-1.50; p = 0.456). For patients eligible for glycemic variability (i.e. at least two in-hospital days and two blood glucose readings), the mortality OR for the SH group adjusted by the same variables as above combined with glycemic variability was 1.21 (95% CI: 0.89-1.64; p = 0.210).

## 4 Discussion

Hospital hyperglycemia is not a problem exclusive to patients with diabetes. In our cohort more than half of the patients with HH were cases of SH with no diagnosis of diabetes. Some patients with hyperglycemia could not be classified as unknown diabetes or SH because there was no previous diagnosis of diabetes and their HbA1c values were missing. These patients were excluded from our study to avoid misclassification. Possibly, future studies with prospective evaluation of HbA1c levels may be able to contribute to the classification of these patients.

The overall mortality rate in our cohort (7.61%) is similar to that found in studies describing patients with HH [28, 29]. In the group of patients diagnosed with diabetes, we found a higher incidence of oncohematological and cardiovascular comorbidities, although adjusted in-hospital mortality did not differ between the two groups. A probable interpretation could be that patients with diabetes have more comorbidities, but stress hyperglycemia reflects a pathology of greater in-hospital severity.

Female sex, stay in ICU or CCU during hospitalization, undergoing surgery, and hospital stay >7 days were associated significantly with SH, reflecting all the potential causes of stress that may lead to hyperglycemia. Remarkably, this association was not found after multivariate analysis and not in unscheduled hospitalizations, which could also be considered a stressor.

The frequency of blood glucose recordings obtained from venous and capillary monitoring was higher in the SH group than in the HH-DBT group (8 versus 6 values; p < 0.01), which may have contributed to the lower glycemic values, better glycemic goals, and lower hypoglycemic events observed in the SH group. Similar results were observed in another patient cohort from the internal medicine section, where the number of glycemic records was higher in diabetes patients. Also, a higher percentage of hypoglycemia was found in this group [29]. There could be a relationship between the greater number of records and better glycemic control with a lower subsequent rate of hypoglycemia in the SH group. The rate of hypoglycemia in this cohort (9.38% total and 1.53% severe) was similar to that in a study published in 2015 in relation to Spanish hospitals [30].

When exploring glycemic variability, we found no difference between the patient groups by using the coefficient of variability. We did not find an association between glycemic variability and longer hospital stay, even though this association has been previously described in the literature [31]. In both groups, glycemic variability measured by the percentage of the coefficient of variability was considered low as it was less than 33.5% [27].

When analyzing insulin treatment, we found that there was less use of the three insulin schemes in patients with HH. In both groups, the most frequently used insulin treatment was the bolus and not the basal-bolus scheme, which is recommended by current guidelines [32].

One of the major limitations of this study lies in the use of retrospective data, which has certain limits. Other covariables and potential confounders related to glycemic control (such as changes in diet, medication, or the clinical status of patients) were not considered because such data could not be reliably retrieved from the records. For the same reason, several comorbidities such as obesity-induced hyperglycemia were not taken into consideration. Also, the study was carried out in our center and may not be representative of the population in other hospitals.

To the best of our knowledge, this is the first study that compares and describes glycemic control and mortality in a cohort of patients with hospital hyperglycemia in South America, classifying patients by whether or not they have diabetes.

## 5 Conclusions

Hospital hyperglycemia is a common problem, not exclusive to diabetes patients. In our cohort, patients with SH had fewer comorbidities and better glycemic control than patients with HH and diabetes. However, there was no difference in hospital mortality between the groups. This could highlight the importance of SH as a risk factor of morbidity and mortality in hospitalized patients.

## Abbreviations

CCU: coronary care unit

CDR: clinical data repository

CoV: coefficient of variation

EH: electronic health records

GG: glycemic goal

HbA1c: glycated hemoglobin

HH-DBT: hospital hyperglycemia with previously diagnosed diabetes

HH: hospital hyperglycemia

ICU: intensive care unit

IQR: interquartile range

OR: odds ratios

SD: standard deviation

SH: stress-induced hyperglycemia
